# Infectious Disease: ExPECting the Worst

**Published:** 2005-06

**Authors:** Melissa Lee Phillips

Dramatic media reports have alerted the public to the dangers of foodborne pathogens such as *Salmonella* and *Escherichia coli* O157:H7. But less-publicized microbes may soon become serious public health threats as well. Two foodborne bacteria, extraintestinal pathogenic *E. coli* (ExPEC) and antimicrobial-resistant *Campylobacter*, are becoming more prevalent, according to studies published in the 1 April 2005 *Journal of Infectious Diseases*.

ExPEC causes millions of urinary tract infections and an estimated 36,000 sepsis deaths each year in the United States alone, and untold numbers globally. ExPEC can live in the gut, but—unlike other classes of *E. coli*—causes infection only if it travels to other parts of the body. ExPEC can live in the intestine for weeks without causing symptoms before inducing illness elsewhere in the body, says Kirk Smith, supervisor of the Foodborne, Vectorborne, and Zoonotic Disease Unit of the Minnesota Department of Health. This delay in onset can create the illusion that an infection is caused by something other than a foodborne pathogen.

In the first *Journal of Infectious Diseases* paper, Smith and colleagues at the University of Minnesota–Twin Cities report their analysis of *E. coli* contamination in foods they bought at 10 Minneapolis–St. Paul markets between 2001 and 2003. They found *E. coli* in 24% of the 1,648 items sampled, including 92% of poultry items, 69% of beef and pork items, and 9% of ready-to-eat foods such as produce, cheeses, and delicatessen items. Almost half of the *E. coli* found in poultry products was ExPEC; about one-fifth of the *E. coli* from beef and pork and a small percentage of that from ready-to-eat foods was ExPEC.

The number of *E. coli* organisms found in each food sample was relatively low, says Sita Tatini, a professor emeritus of food science and nutrition at the University of Minnesota and senior author of the paper. However, ExPEC’s virulence factors—the properties that permit it to infect tissue—allow even a small number of bacteria to cause disease, Tatini says.

The scientists also found that 94% of poultry samples contaminated with *E. coli* contained a strain that was resistant to at least one antibiotic. They isolated resistant strains from 85% of *E. coli*–contaminated beef and pork and from 27% of *E. coli*–contaminated ready-to-eat items.

The second paper focused on drug-resistant strains of *Campylobacter*. Kåre Mølbak, director of the Department of Epidemiology at the Statens Serum Institut in Copenhagen, examined the clinical effects of human infection with *Campylobacter* strains resistant to quinolones and erythromycin.

By accessing the Danish government’s national registry of patient admissions and discharges, Mølbak and his colleagues were able to track the outcomes of about 3,500 people who were diagnosed with *Campylobacter* infections between 1996 and 2000. Within 30 days of infection, patients with quinolone-resistant infections were more than six times as likely as patients infected with susceptible strains to die or suffer an invasive illness such as meningitis, abscess, pancreatitis, or hepatitis. Within 90 days of infection, patients with erythromycin-resistant infections were more than five times as likely to die or to be diagnosed with an invasive illness.

Antibiotic overuse by people is just one reason why we’re now seeing more antibiotic-resistant microbes, says Wondwossen Abebe Gebreyes, an assistant professor of food safety and molecular epidemiology at North Carolina State University in Raleigh. Farmers in many countries use antibiotics not only to treat or prevent infection but also to promote growth of healthy animals. Fluoroquinolones have been used in human medicine since the 1980s, but it was not until farmers began to use them to treat animal infection in the 1990s that resistant bacterial strains appeared. In some countries, quinolone-resistant *Campylobacter* species are now more common than quinolone-susceptible strains.

In Denmark, the prevalence of *Campylobacter* species resistant to macrolide-class antibiotics such as erythromycin has dropped since 1998, when all growth promoters, including macrolides, were banned from use in livestock. “That’s really good news,” says Mølbak, “because that suggests that if you change the policy—for example, improve hygiene and management practices rather than give the animals antibiotics—then you are able to reverse the situation.” Use of fluoroquinolones is limited but not banned in Danish livestock.

Indeed, in most countries, antibiotic use on farms is on the rise, and so is antibiotic-resistant bacterial infection in humans, says Martin Blaser, chair of medicine at New York University and president-elect of the Infectious Diseases Society of America. Resistance has “been recognized as a cost of antibiotic use for more than fifty years,” Blaser says. “As a society, we’re using more and more [antibiotics], so it’s not surprising that resistance is growing.”

